# Supplementary material for: MED1 Ablation Promotes Oral Mucosal Wound Healing via JNK Signaling Pathway
Source: Int J Mol Sci. 2022 Nov 2;23(21):13414. doi: 10.3390/ijms232113414 (PMC9655393; doi:10.3390/ijms232113414)
Supplement: Supplementary file 1 [file ijms-23-13414-s001.zip › ijms-1948245-supplementary.pdf]

Supplementary Materials:

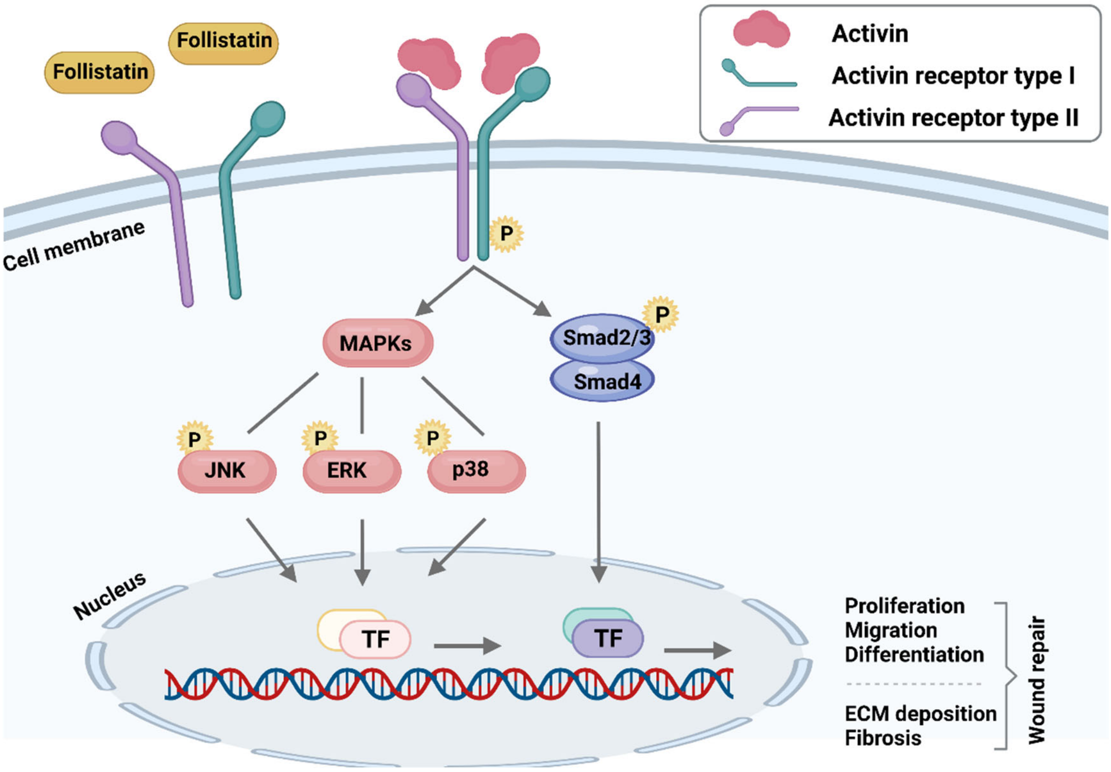

Figure S1. Activin signaling pathway. Activin receptor (ActR) belongs to serine/threonine kinase type receptor. ActR can be divided into ActRI and ActRII. Activins bind to ActRII and activate them. The activated ActRII can further recruit ActRI and phosphorylated ActRI, which can activate two signaling pathways: the Smad signaling pathway and the MAPK signaling pathway.

Table S1. Primers used in this study.

Primer	Sequence (5′-3′)
GAPDH	F: ACAACTTTGGTATCGTGGAAGG<br>R: GCCATCACGCCACAGTTTC
MED1	F: GAGGGCATCAACATTTGGTCA<br>R: AGATGAGAGCCCAGTCCATTC
Follistatin	F: AGGCAAGATGTAAAGAGCAGC<br>R: CAGTAGGCATTATTGGTCTGGTC
Activin A	F: ACGGGTATGTGGAGATAGAGGA<br>R: GGACTTTTAGGAAGAGCCAGACT
JNK	F: TGTGTGGAATCAAGCACCTTC<br>R: AGGCGTCATCATAAAACTCGTTC
c-Jun	F: TCCAAGTGCCGAAAAAGGAAG<br>R: CGAGTTCTGAGCTTTCAAGGT

Table S2. The antibodies used in the Western Blot.

Type	Antibody name and catalogue number	Working dilution	Company
Primary antibody	anti-β-actin (YT0099)	1:1500	Immunoway Biotechnology (USA)
	anti-MED1 (ab245546)	1:2000	abcam (UK)
	anti-Follistatin (sc365003)	1:200	Santa Cruz Biotechnology (USA)
	anti-Activin A (ab89307)	1:1000	abcam (UK)
	anti-JNK (ab179461)	1:4000	abcam (UK)
	anti-phospho-JNK (#4668)	1:1000	Cell Signaling Technology (USA)
	anti-c-Jun (ab32385)	1:2000	abcam (UK)
	anti-phospho-c-Jun (#3270)	1:1000	Cell Signaling Technology (USA)
Secondary antibody	anti-mouse IgG (RS0001)	1:2500	Immunoway Biotechnology (USA)
	anti-rabbit IgG (RS0002)	1:2500	Immunoway Biotechnology (USA)
